# The New Party’s Platform; the Public Health

**Published:** 1912-09

**Authors:** 


### Abstracts and Selections.

THE NEW PARTY'S PLATFORM; THE PUBLIC HEALTH.—"We favor the union of all the existing agencies of the federal government dealing with the public health into a single national health service, without discrimination against or for any one set of therapeutic methods, school of medicine or school of healing, with such additional powers as may be necessary to enable it to perform efficiently such duties in the protection of the public from preventable disease as may be properly undertaken by the federal authorities, including the execution of existing laws regarding pure food, quarantine and cognate subjects, the promotion of appropriate action for the improvement of vital statistics and the extension of the registration area of such statistics, and co-operation with the health activities of the various States and cities of the nation."

